# The Drunkard’s Nose

**Published:** 1886-03

**Authors:** 


### THE DRUNKARD'S NOSE.

One of the most beautiful features of the face is a shapely, fine-curved nose. If this be deformed, the whole face is injured, however perfect otherwise. But a bad nose is the portion of every habitual tippler. It takes on a hated red (more intense as the years go on), becomes coarse with pimples, or swells out with disgusting and livid protuberances—"toddy blossoms," is the apt and picturesque language of the common people. The tippler may try ever so hard to conceal his habits, but his nose is an emblazoned signal, proclaiming the fact to every new comer.

The explanation is this: The alcohol increases the action of the heart and arteries about one-fifth, thus driving the blood to the surface faster than the veins can bring it back. Hence the countless capillaries, whose minuteness makes them normally invisible, are distended with impure blood, are kept in a state of permanent congestion, and give rise to pimples and blotches.

But the nose is not alone in its dishonor and suffering. Every organ of the body is in a similar condition. The head therefore aches; the sleep is disturbed; the appetite is poor; the liver is disordered; the tongue is coated; the throat is dry; the heart has spells of palpitation; the back and limbs suffer frequent pains; and the lungs become inflamed from the slightest exposure. This is not a mere deformity, nor simply a prominent sign of a degrading habit; it is a note of warning to its possessor that his whole system is diseased, and is getting ready for a drunkard's grave.

Says the *Medical Reporter:* "It is a medical fact that as the influence of alcohol reddens the dram-drinker's nose, and changes its appearance, so it reddens and changes the appearance of every organ of the body; and as the nose thus affected is not in a natural or healthy condition, so every organ of his body is changed from a natural and healthy condition to an unnatural and diseased condition; and as the skin of the nose takes on unhealthy action, so the substance and coverings of the internal organs take on diseased action, which results in the full development of incurable diseases, such as insanity, diseases of the heart, Bright's disease of the kidneys, hob-nail liver, and slow inflammation of the stomach. All these diseases exist at the same time in the dram drinker, but the organ most diseased is apt to take the lead in the morbid action."

---

**The** *Southern California Practitioner* says: "We believe that the most effectual plan the regular medical profession can adopt to maintain their supremacy, is by working to deserve that supremacy."

